# Prostate-specific antigen-based screening in Afro-Caribbean men: a survey of members of the Caribbean Urological Association

**DOI:** 10.3332/ecancer.2018.842

**Published:** 2018-06-11

**Authors:** Satyendra Persaud, William D Aiken

**Affiliations:** 1 Division of Clinical Surgical Sciences, University of the West Indies, Trinidad and Tobago; 2 Department of Surgery, Radiology, Anaesthesia and Intensive care, Section of Surgery, Division of Urology, Faculty of Medical Sciences, University of the West Indies, Mona Campus, Kingston, Jamaica

**Keywords:** prostate cancer, prostate-specific antigen screening, Afro-Caribbean, Caribbean, urologists

## Abstract

**Objectives:**

To examine the attitudes, beliefs and practices of Caribbean urologists regarding prostate-specific antigen (PSA)-based screening in the Caribbean region particularly as it relates to Afro-Caribbean men.

**Design:**

An Internet-based descriptive cross-sectional study using a standardised questionnaire designed to capture information on respondents’ attitudes, beliefs and practices towards PSA-based screening was conducted using the online survey tool Survey Monkey among known urologists in the Caribbean, based on the complete mailing list of the membership of the Caribbean Urological Association.

**Results:**

Thirty of the total population of 40 urologists (75%) from nine countries in the Caribbean completed the survey. Twelve (40%) were from Jamaica and eight (26.7%) were from Trinidad. Two-thirds (*n* = 20) of the urologists believed that PSA-based screening has positively impacted survival in their population and 76.7% (*n* = 23) supported the PSA-based screening in the Afro-Caribbean male. Seventy-eight percent believed that guidelines from other countries were not applicable to the Caribbean and 63% believed that a regional body should publish its own guidelines. Most supported yearly screening with the PSA and digital rectal examination beginning at age 40 for Afro-Caribbean men but opinion varied regarding PSA-based screening of Indo-Caribbean men. Respondents were unanimous in their belief that there should be an upper age limit for screening, 75 years old being the most commonly reported.

**Conclusion:**

Most Caribbean urologists favour PSA-based screening in Afro-Caribbean men and recommend that Caribbean-specific guidelines need to be drafted.

## Introduction

Prostate cancer is a serious public health problem in the Caribbean with prostate-cancer-specific mortality rates being the highest in the world [1]. Men of African descent in new world settings such as the Caribbean and North America are disproportionately affected by prostate cancer with higher incidence and mortality rates. Most territories in the Caribbean have a predominantly black population and in those with an almost equal mix of persons of African descent and Indo-Asian extraction such as in Trinidad, the incidence and mortality rates are three times greater among blacks compared to Indians [2, 3].

Secondary prevention of prostate cancer through prostate-specific antigen (PSA)-based screening has been done on an ad hoc basis in the Caribbean by private practitioners, urologists and cancer societies since PSA was introduced into clinical practice in 1988 but despite this, almost 50% of men still present with symptomatic, locally advanced and metastatic disease in territories such as Jamaica [4]. There has been no documented downward stage migration in incident cases of prostate cancer in the Caribbean and this is likely to be due to the poor uptake and an ad hoc nature of PSA-based prostate cancer screening. Added to this is the uncertainty and controversy surrounding the efficacy of PSA-based screening in decreasing prostate-cancer-specific mortality while minimising harms associated with screening.

Randomised clinical trials involving mostly Caucasian participants such as the prostate lung, colon and ovarian (PLCO) study have demonstrated only a modest reduction in prostate-cancer-specific mortality from screening while demonstrating a significant risk of over-detection and possible overtreatment [5]. This has made the PSA screening a controversial issue and had prompted the United States Preventive Services Task Force (USPSTF) to initially recommend against screening, stating that the harms associated with screening outweigh the benefits [6]. However, the USPSTF has recently softened its stance on screening [7].

Doubts have been expressed by Caribbean urologists regarding the external validity of international guidelines when applied to predominantly black populations, such as that of the Caribbean [8], in which the biology of prostate cancer may be more aggressive when compared with Caucasians [9] and in which almost 50% of men present with advanced disease [4], factors which no doubt contribute to the Caribbean having the highest prostate-cancer-specific mortality in the world [10]. This has prompted calls by some Caribbean urologists for continued PSA-based screening in the Caribbean until clinical trials specifically targeting black populations with high prostate cancer incidence and mortality study the efficacy of PSA-based screening among them [11]. As there is a divergence of opinion regarding PSA-based screening among different medical organisations and practitioners, this study was designed to determine the attitudes, beliefs and practices of Caribbean urologists regarding PSA-based screening in their populations, populations known to be at high risk of death from prostate cancer.

## Methods

Between 1 August and 30 November 2013 all English-speaking Caribbean urologists as well as Urologists from Haiti who are members of the Caribbean Urological Association (CURA) and on its mailing list were sent invitations via email to participate in an online survey using the survey tool Survey Monkey. A standardised questionnaire was designed to capture information on demographic data as well as respondent’s attitudes, beliefs and practices regarding PSA-based screening. With the exception of a few urologists in Trinidad and Tobago as well as Barbados, most urologists in the English-speaking Caribbean are members of the CURA and on its mailing list. There were no exclusion criteria and all urologists listed, regardless of whether they were paid up or not, were invited to participate. The process was entirely voluntary. Data were compiled and analysed using the STATA version 7.

## Results

**Figure 1.**
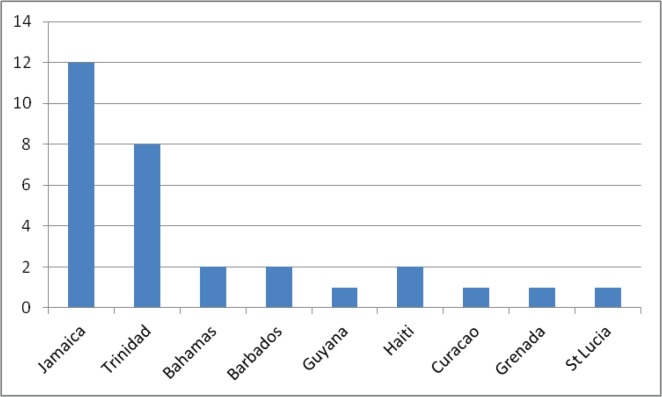
Distribution of respondents by country.

Seventy-five percent (30 of 40) of questionnaires that were distributed were answered and returned electronically. In total, nine Caribbean territories were represented (Figure 1), with Jamaica (12) and Trinidad and Tobago (8) having the highest number of urologists. The average age of the respondents was 49.7 years (SD ± 11.2 years). Most of the respondents did their urological training either in the Caribbean (36.7%) or in the United Kingdom (36.7%).

### Support for PSA screening

Ninety-three percent (28 of 30) of respondents indicated that Afro-Caribbean men constituted the majority of their patient population. The majority of urologists were of the opinion that PSA-based screening had positively impacted survival in their patient population and 76.7% were in support of PSA-based screening (Tables 1 and 2).

### The screening process

Sixty-six percent (18 of 27) believed that PSA screening in Afro-Caribbean men should commence at age 40 and respondents were unanimous in their view that there should be an upper age limit for screening. The upper age limit most frequently selected by respondents was 75 years (55.5%). Opinions varied as to whether this should be adjusted for men of different races, for example, East Indians, with 48 % (13 of 27) saying yes and 52% (14 of 27) saying no. Seventy-eight percent of respondents felt that screening should be annual rather than biennial while 93% felt that the digital rectal examination (DRE) should be a part of the screening protocol.

Half of the urologists surveyed indicated that they always discussed the pros and cons of PSA screening with the patient prior to ordering the test (Table 3). All urologists felt that patients understood at least some of the screening discussion (Table 4).

### Validity and future of PSA screening

Seventy-eight percent (21 of 28) of respondents felt that international screening guidelines were not applicable to the Caribbean (Table 5). Sixty-three percent (17 of 27) believed that a multinational committee, for example, the Caribbean urological Association, should formulate the Caribbean-specific guidelines while 30% (8 of 27) felt that guidelines should be drafted at a local level. Seven percent stated that individual urologists should choose which guidelines they would like to follow (Table 6).

When asked about the future of PSA screening in the region, 64% (18 of 28) of respondents opined that PSA screening in the Caribbean will increase, 14% (4 of 28) felt that it will decrease and 21% (6 of 28) felt that it will remain unchanged (Table 7).

## Discussion

This paper, as far as the authors are aware, represents the first time that an attempt has been made to solicit the opinions of regional urologists as a group. We have found that the majority (77%) of urologists surveyed were supportive of PSA screening. Widespread support for PSA screening is not surprising in the context that prostate cancer mortality in the Caribbean are among the highest in the World [1, 10]. There is some evidence which may support the notion that PSA screening may be impactful in the Caribbean region. In a recent review, the authors noted that compared to Afro-American men, Afro-Caribbean men were diagnosed later and had worse 5-year survival rates (41.6% versus 84.4%). Interestingly, there was no difference in 5-year survival between the immigrant Caribbean men and African American men. The authors suggest that later diagnosis among Caribbean men may be to blame for the higher mortality noted and recommended interventions aimed at earlier detection [12]. Similar findings were noted when Jamaican men were compared to their African-American counterparts in Chicago [13].

Overall, 77% of urologists were in support of PSA screening among Afro-Caribbean men. Among the 10% who did not support screening, the most common reason cited was a ‘lack of evidence’ to support it, with respondents most commonly referencing the initial USPSTF recommendation. The US Preventative Services Taskforce based this recommendation largely on review of two clinical trials, one American and the other European [5, 14]. Not only did these studies, particularly the PLCO Trial, have significant limitations, not least of which was cross contamination which would have biased the results towards the null, but one may question the extrapolation of their conclusions to a Caribbean population. For example, African-Americans comprised only 4.5% of those enrolled in the PLCO Trial [5]. This disproportional representation limits our ability to extrapolate the results of this study to our largely Afro population which varies by territory, ranging from 50% to 93%. Indeed, more than three quarters of urologists surveyed believed that the international guidelines were not applicable to the Caribbean and there have been calls from regional experts for Caribbean territories to consider the feasibility of screening [11]. Although the Jamaican urological society has issued guidelines for Jamaica, none have been issued for the region as a whole—in our survey two-thirds were of the opinion that a regional body would be the best suited to issue guidelines.

Sixty-six percent of respondents believed that screening in the black male should commence at age 40. In an earlier study targeting medical consultants in Jamaica, 85% believed that prostate cancer screening should begin at 40 [15]. Guidelines have been issued by the Jamaican Urological Society–Jamaican men 40 years and older with a life expectancy of at least 10–15 years are advised to undergo annual PSA screening and DRE [16]. Yet in a survey, only 35% of the Jamaican men reported having been screened [11]. Several factors may account for this including poor access to care, lack of doctor-patient rapport on the subject and the stigma associated with the DRE.

While it was noted that most urologists do have dialogue with their patients prior embarking on screening, there is still room for improvement as almost a third felt that the patient understands only some of what is relayed during the discussion. This is the reason for the concern as a thorough discussion with the patient is a central theme in the guidelines recently issued by several major urological organizations [17, 18].

Although the majority of Caribbean urologists were in support of screening Afro-Caribbean men, they were divided on whether screening guidelines should be modified for men of other races. Those who advocated for a race-based modification in screening most commonly suggested commencing screening at an older age or increasing the screening interval, for example, from yearly to biennially.

Based on our survey, if a new Caribbean guideline was to be formulated it would likely read as follows: ‘For Afro-Caribbean men, we recommend yearly screening with PSA and DRE starting at age 40 and stopping at age 75. For men of other races, consider commencing screening later and/or increasing the interval between screenings’. Although the opinion of each urologist is likely informed by his or her interpretation of available evidence, this guideline statement would be based on expert opinion.

However, our work was not without limitations. While every attempt was made to contact as many urologists as possible, and an overall response rate of 75% was good, we are aware that we were unable to reach everyone. A good example would be Haiti where the two urologists surveyed represented just a fraction of the total number of practising urologists—these urologists are currently not on the mailing list of the CURA. Also, our findings on how to deal with the Indo-Caribbean men or Caucasians were equivocal and perhaps further work on this may be warranted. Primary care physicians are the point of first contact for many men potentially seeking to be screened and their views on screening are critical in framing a discussion with the patient. We therefore feel that this demographic should be a target for a future project similar to the one we have carried out.

## Conclusions

In the Caribbean, prostate cancer is a significant public health concern as the incidence and mortality in the region are among the highest in the world. However, no guidelines exist for the region as a whole. This paper confirms that urologists in the Caribbean are in support of PSA-based screening among the Afro-Caribbean men and many feel region-specific guidelines should be drafted.

## Conflicts of interest and funding

The authors have no conflicts of interest to declare and received no funding of any kind for the production or publication of this paper.

## Figures and Tables

**Table 1.** Perceptions about the impact of screening.

Do you believe, based on your experience, that PSA screening has positively impacted survival in your patient population?		
Answer options	Response %	Response count
Yes	66.7%	20
No	20.0%	6
Don’t know	13.3%	4
Answered question		30
Skipped question		0

**Table 2.** Support for screening Afro-Caribbean men.

Do you currently support PSA screening in the asymptomatic Afro-Caribbean male?		
Answer options	Response %	Response count
Yes	76.7%	23
No	10.0%	3
Undecided	13.3%	4
Answered question		30
Skipped question		0

**Table 3.** Obtaining consent for screening.

On average, how often do you fully discuss the pros and cons of PSA screening with a patient (or proxy) prior to ordering a PSA test?		
Answer options	Response %	response count
Always	50.0%	14
>50% of the time	32.1%	9
<50% of the time	7.1%	2
Rarely	7.1%	2
Never	3.6%	1
Answered question		28
Skipped question		2

**Table 4.** Perceptions of patient comprehension.

Do you feel that the typical patient/proxy understands the discussion on screening?		
Answer options	Response %	response count
Most of what is said	35.7%	10
Some of what is said	64.3%	18
None of what is said	0.0%	0
Answered question		28
Skipped question		2

**Table 5.** Applicability of International guidelines.

Do you believe that international screening guidelines are applicable to the Caribbean?		
Answer options	Response %	Response count
Yes	22.2%	6
No	77.8%	21
Answered question		27
Skipped question		3

**Table 6.** The need for Caribbean screening guidelines.

Do you believe that the Caribbean should have its own screening guidelines?		
Answer options	Response %	Response count
No, each urologist should choose what guideline he/she wants to follow	7.4%	2
Yes, each country should formulate its own guidelines	29.6%	8
Yes, a multinational committee under the auspices of a regional body (e.g. CURA) should lead	63.0%	17
Other (please specify)		0
answered question		27
skipped question		3

**Table 7.** The future of PSA screening in the region.

What do you foresee as the future of PSA screening in the Caribbean?		
Answer options	Response %	Response count
It will increase	64.3%	18
It will decrease	14.3%	4
It will remain unchanged	21.4%	6
Answered question		28
Skipped question		2
